# Age-Related Disparities in Stroke Knowledge Among Community Older Adults

**DOI:** 10.3389/fneur.2021.717472

**Published:** 2021-09-08

**Authors:** Xiaochuan Liu, Huiwen Gui, Sichen Yao, Zhongcheng Li, Jing Zhao

**Affiliations:** ^1^Department of Neurology, Minhang Hospital, Fudan University, Shanghai, China; ^2^Wujing Community Health Service Center, Shanghai, China

**Keywords:** older adult, stroke awareness, community resident, oldest old, aging

## Abstract

**Background:** This study aimed to investigate the disparities in stroke knowledge between older adults and the oldest old.

**Methods:** Family physicians conducted a cross-sectional survey through face-to-face interviews with the older and oldest old adults of two suburban communities in the Minhang district, Shanghai between October 1, 2020, and November 30, 2020. All participants were classified as oldest old (age ≥80 years) and older adults (age 60–79 years). Between-group differences in stroke knowledge were investigated by multivariate logistic regression analysis.

**Results:** Overall, 466 older adults including 101 (21.67%) oldest old persons were qualified. Older adults were more familiar with the risk factors and symptoms of stroke than the oldest old. By multivariable logistic regression analysis, older adults were more familiar with the following risk factors: smoking [odds ratio (OR) 0.32, 95% confidence interval (CI) 0.16–0.61], alcohol abuse (OR 0.45, 95% CI 0.23–0.87), dyslipidemia (OR 0.51, 95% CI 0.31–0.85), and obesity (OR 0.30, 95% CI 0.17–0.53) than the oldest old. Regarding stroke symptoms, older adults were more aware regarding vision alteration (OR 0.42, 95% CI 0.25–0.69) and face-drop (OR 0.57, 95% CI 0.35–0.95) than the oldest old. The oldest old were less aware of acute stroke therapy (OR 0.11, 95% CI 0.02–0.48) and calling the emergency medical service (OR 0.30, 95% CI 0.12–0.70) than older adults. Finally, the older adults used television (OR 0.53, 95% CI 0.28–1.0), WeChat (OR 0.21, 95% CI 0.05–0.89), and the community bulletin board (OR 0.43, 95% CI 0.23–0.80) as knowledge sources more than the oldest old.

**Conclusion:** The older adults and the oldest old had significantly high disparities in stroke knowledge. Given the aging population across China, the life expectancy is expected to be longer in future decades. These differences should be addressed in stroke educational campaigns targeting the oldest old.

## Introduction

The seventh Chinese census revealed that population aging is a growing concern that requires the government's keen attention (1). There are 264 million people aged >60 years, which is 5.44% higher than the older population from 10 years ago. Population aging not only challenges China but also the rest of the world. The proportion of older adults aged >75 years has significantly increased and is expected to increase by >10% in developed countries by 2050 (2).

Meanwhile, it is well-known that the stroke risk increases with age, with the highest number of life-threatening events affecting the elderly population. Moreover, previous studies showed a strong correlation between aging and poor stroke-related awareness, as older adults acquire limited stroke knowledge (3–7). About one-third of patients with stroke are aged >80 years; moreover, their risk of fatalities is higher than patients aged <80 years without stroke (8). The oldest old (aged >80 years) have seldom been investigated regarding stroke awareness, and poor stroke awareness is not much comparable between adults aged >80 years and 60–80 years. Hence, this study aimed to explore the disparities in stroke knowledge among community adults within these two age groups.

## Methods

### Study Design and Population

Between October 1, 2020 and November 30, 2020, a cross-sectional survey was conducted by family physicians and their team members through face-to-face interviews with older residents of two suburban communities in Minhang District, Shanghai, by random selection. The survey was conducted during visits by community-dwelling older adults for medical advice from family physicians regarding the management of chronic diseases, including hypertension and diabetes. We enrolled community residents aged ≥60 years who lacked acute stage cardiovascular diseases. In each household, we only invited one family member who was eligible to complete the survey. We excluded residents who refused to participate after receiving descriptions of the study objectives. The included participants were classified as the oldest old (age ≥80 years) and older adults (age: 60–79 years). The survey had three parts, namely, basic information, medical history, and stroke knowledge (survey questions and other details available in the online Supplementary Material). Briefly, the participants were inquired regarding the awareness of stroke symptoms and risk factors, the immediate actions necessary upon identifying a stroke, and tools for acquiring stroke knowledge. For example, interviewers would ask participants whether they would consider someone who suddenly complains about trouble seeing in one or both eyes as having a stroke. Participants responding “no” or “I do not know” were considered not to know the stroke symptom. Regarding the stroke awareness tools, we included two popular and widely used stroke educational tools: Stroke 120 and FAST (Face, Arm, Speech, Time) (9–12). The participants were questioned whether they had heard previously about them and if they could explain their meaning.

The study protocol was approved by the institutional review board affiliated with Fudan University. All participants provided written informed consent before participation. Original data can be shared under reasonable request by contacting the corresponding author.

### Data Collection

There were ~515 households with family members aged ≥60 years living in two communities. Therefore, we were required to interview ≥464 participants to ensure 90% coverage of the population. Family physicians and their team members underwent training by stroke physicians before conducting the face-to-face interviews to avoid inconsistency in the interview script applied in the survey. Prior medical history was determined for each participant based on standard definitions and medical records from local tertiary hospitals.

### Sensitivity Analysis

Since there was no definite cut-point for defining the oldest old, we further analyzed differences in stroke knowledge among community older adults aged >75 and <75 years.

### Statistical Analysis

Statistical analyses were performed on STATA (Version 15.0 Stata Corp College Station, Texas, USA). Categorical variables were analyzed using the chi-squared test or Fisher's exact test, while continuous variables were analyzed using a *t*-test. Among-group differences in stroke knowledge were investigated using logistic regression analysis. We adjusted for potential confounders (gender, educational background, smoking history, drinking habit, hypertension, diabetes, myocardial infarction, prior stroke, dyslipidemia, cancer history, and atrial fibrillation) in multivariable logistic regression analysis. Forest-plot was drawn in R version 3.6.1 (The R Foundation for Statistical Computing). Statistical significance was set at a two-tailed *p*-value < 0.05.

## Results

### Characteristics of All Participants

A total of 511 older adults were invited to respond to the survey. The response rate was 91.19% (15 refused to participate, 20 could not complete the survey independently and were accompanied by their children, and 10 decided to drop out during the survey). Finally, we included 466 older adults without missing information in all surveys. Table 1 shows the characteristics of the older adults [mean age, 73.45 years; 101 (21.67%) persons aged ≥80 years]. The mean age of the oldest-old group was significantly higher than that in the older adult group (age gap: >10 years; *p* < 0.001). Approximately 90% of the oldest old were educated for <6 years, which was a significantly higher proportion than that among older adults aged 60–79 years (*p* < 0.01). Compared with older adults, the oldest old had significantly more ex-smokers (*p* = 0.01). The oldest old had higher proportions of prior medical history [hypertension, myocardial infarction (MI), and atrial fibrillation (AF)] (76.2 vs. 62.2%, *p* < 0.01; 7.9 vs. 2.2%, *p* = 0.01; and 4.0 vs. 0.8%, *p* = 0.04, respectively) than the older adults. However, the older adults had an almost 2 fold higher incidence of diabetes than the oldest old (*p* < 0.001). Generally, there was no between-group difference in the distribution of the incidence of chronic diseases, with hypertension and dyslipidemia being ranked first and second place, respectively.

**Table 1:** Baseline characteristics of the participants in our study.

**Factors**	**All participants**	**Older adult**	**Oldest old**	***p*-value**
***N***	**466**	**365**	**101**	
Age, mean (SD)	73.45 (6.88)	70.82 (5.14)	82.96 (2.73)	<0.01
Age range	60–93	60–79	80–93	
Gender				0.22
Male	215 (46.14%)	174 (47.7%)	41 (40.6%)	
Female	251 (53.86%)	191 (52.3%)	60 (59.4%)	
Educational background				<0.01
Primary school	250 (53.65%)	165 (45.2%)	85 (84.2%)	
Secondary school	181 (38.84%)	169 (46.3%)	12 (11.9%)	
High school	33 (7.08%)	30 (8.2%)	3 (3.0%)	
College degree	2 (0.43%)	1 (0.3%)	1 (1.0%)	
Smoking habit				0.01
Quit	88 (18.88%)	63 (17.3%)	25 (24.8%)	
Never quit	63 (13.52%)	57 (15.6%)	6 (5.9%)	
Never smoke	315 (67.60%)	245 (67.1%)	70 (69.3%)	
Alcohol habit				0.75
Quit	115 (24.68%)	91 (24.9%)	24 (23.8%)	
Never quit	41 (8.80%)	34 (9.3%)	7 (6.9%)	
Never drink	310 (66.52%)	240 (65.8%)	70 (69.3%)	
Medical history				
Hypertension	304 (65.24%)	227 (62.2%)	77 (76.2%)	<0.01
Diabetes	116 (24.89%)	104 (28.5%)	12 (11.9%)	<0.01
Prior MI	16 (3.43%)	8 (2.2%)	8 (7.9%)	0.01
Prior stroke	30 (6.44%)	22 (6.0%)	8 (7.9%)	0.49
Dyslipidemia	154 (33.05%)	114 (31.2%)	40 (39.6%)	0.12
Cancer history	9 (1.93%)	9 (2.5%)	0 (0.0%)	0.22
AF	7 (1.5%)	3 (0.8%)	4 (4.0%)	0.04

*MI, myocardial infarction; AF, atrial fibrillation; P-values represent the differences between the older adults and the oldest old*.

### Awareness of Stroke-Related Knowledge Among the Oldest Old and Older Adults

**Figure 1:**
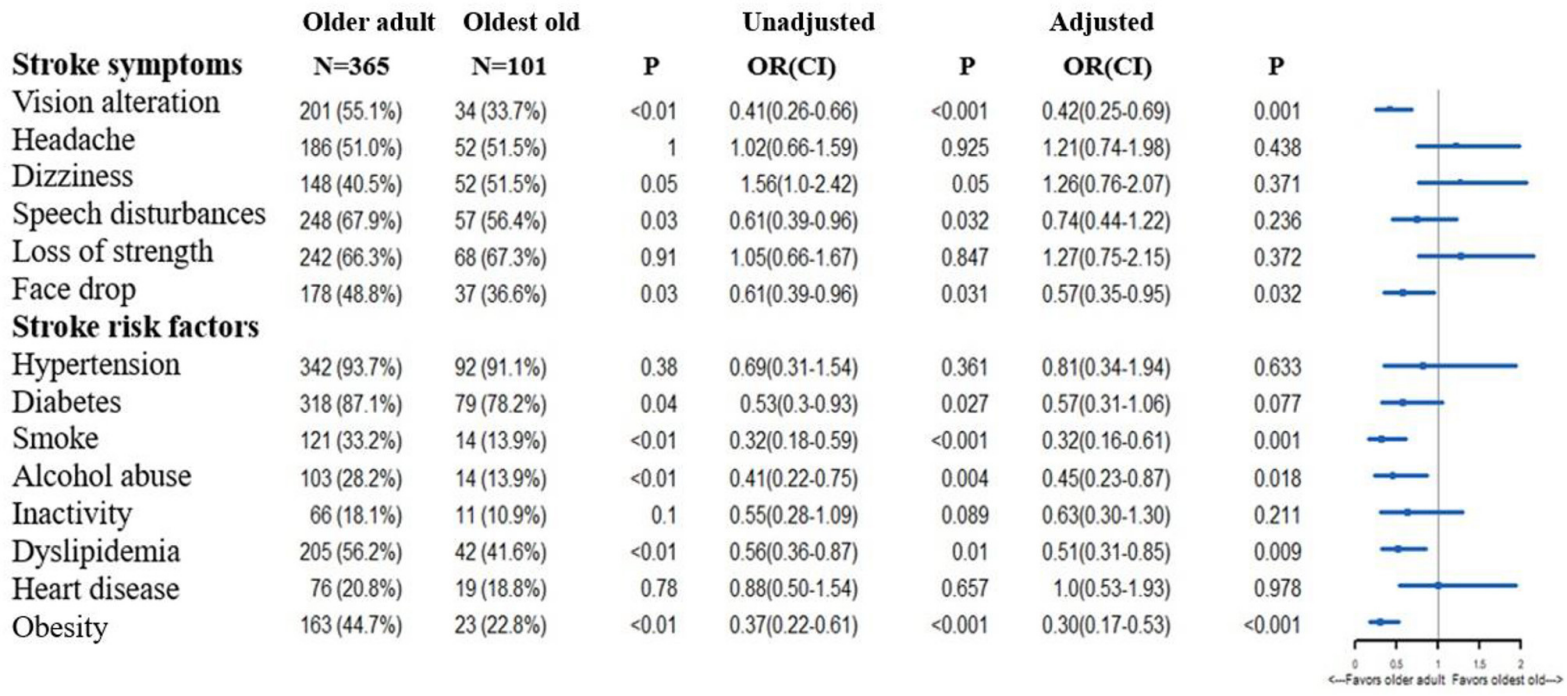
Differences in the knowledge of stroke symptoms and risk factors between older adults and the oldest old. OR, Odds Ratio; CI, Confidence Interval. In the multivariable logistic regression analysis, we adjusted gender, educational background, smoking history, drinking habit, hypertension, diabetes, myocardial infarction, prior stroke, dyslipidemia, cancer history, atrial fibrillation. The OR means the comparison between the older adults and oldest old (oldest old vs. the older adults).

Figure 1 reveals between-group disparities in stroke knowledge. Notably, there was a tendency that older adults were more familiar with stroke risk factors and symptoms than the oldest old. Multivariable logistic regression analysis revealed that older adults were more familiar with smoking [odds ratio (OR) 0.32, 95% confidence interval (CI) 0.16–0.61], alcohol abuse (OR 0.45, 95% CI 0.23–0.87), dyslipidemia (OR 0.51, 95% CI 0.31–0.85), and obesity (OR 0.30, 95% CI 0.17–0.53) as stroke risk factors. Regarding the awareness of stroke symptoms, older adults had a higher awareness of vision alteration (OR 0.42, 95% CI 0.25–0.69) and face drop (OR 0.57, 95% CI 0.35–0.95). Although the oldest old were more familiar with the stroke symptoms of headache, dizziness, and loss of strength, there was no significant between-group difference (all *p* > 0.05).

**Figure 2:**
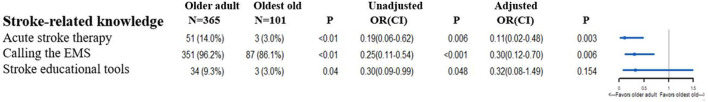
Differences in the awareness of stroke-related knowledge between older adults and the oldest old. OR, Odds Ratio; CI, Confidence Interval; EMS, emergency medical service. Acute stroke therapy included thrombolytic therapy and endovascular therapy. Stroke educational tools included FAST and Stroke 1-2-0. In the multivariable logistic regression analysis, we adjusted gender, educational background, smoking history, drinking habit, hypertension, diabetes, myocardial infarction, prior stroke, dyslipidemia, cancer history, atrial fibrillation. The OR means the comparison between the older adults and oldest old (oldest old vs. the older adults).

There were similar between-group disparities in the awareness of stroke-related knowledge (Figure 2). Older adults tended to have a higher awareness of acute stroke therapy, including thrombolytic and endovascular therapy (OR 0.11, 95% CI 0.02–0.48). Notably, the overall awareness of acute stroke therapy was low in both groups.

**Figure 3:**
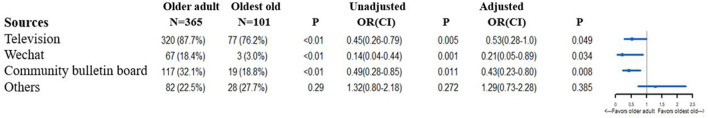
Differences in the sources of acquiring stroke-related knowledge between older adults and the oldest old. OR, Odds Ratio; CI, Confidence Interval. In the multivariable logistic regression analysis, we adjusted gender, educational background, smoking history, drinking habit, hypertension, diabetes, myocardial infarction, prior stroke, dyslipidemia, cancer history, atrial fibrillation. The OR means the comparison between the older adults and oldest old (oldest old vs. the older adults).

Additionally, Figure 2 demonstrates that the awareness of calling the emergency medical service (EMS) was lower in the oldest old (OR 0.30, 95% CI 0.12–0.70). Despite the implementation of FAST and Stroke 120 as stroke promotion tools in numerous stroke educational campaigns, <10% of the older adults were aware of them. Figure 3 reveals between-group differences in the sources of acquiring stroke-related knowledge. Regarding the sources of learning stroke-related knowledge, television (OR 0.53, 95% CI 0.28–1.0), WeChat (OR 0.21, 95% CI 0.05–0.89), and community bulletin board (OR 0.43, 95% CI 0.23–0.80) were more popular among older adults than among the oldest old. Among the three sources, television was the most popular source for each group.

### Sensitivity Analysis

As aforementioned, the cutoff for the oldest old and older adults is not known; therefore, we analyzed between-group differences in stroke knowledge considering 75 years age as the cut-off. Table 2 shows the characteristics of the two groups. There were more females among the oldest old (*p* = 0.04). The oldest old had higher proportions of prior medical history, including hypertension and dyslipidemia (80.2 vs. 56.1%, *p* < 0.01; 41.2 vs. 28.0%, *p* < 0.01, respectively) than the older adults. However, compared with the oldest old, older adults had higher prevalence of diabetes (*p* = 0.05). Generally, the distribution of the prevalence of chronic diseases was similar as the age cutoff of 80 years.

**Table 2:** Baseline characteristics of the community older adults (age cut-off 75 years).

**Factors**	**Older adult**	**Oldest old**	***p*-value**
***N***	**289**	**177**	
Age, mean (SD)	69.05 (4.23)	80.64 (3.46)	<0.01
Age range	60–75	76–93	
Gender			0.04
Male	144 (49.8%)	71 (40.1%)	
Female	145 (50.2%)	106 (59.9%)	
Educational background			<0.01
Primary school	107 (37.0%)	143 (80.8%)	
Secondary school	152 (52.6%)	29 (16.4%)	
High school	29 (10.0%)	4 (2.3%)	
College degree	1 (0.3%)	1 (0.6%)	
Smoking habit			<0.01
Quit	51 (17.6%)	37 (20.9%)	
Never quit	50 (17.3%)	13 (7.3%)	
Never smoke	188 (65.1%)	127 (71.8%)	
Alcohol habit			0.10
Quit	77 (26.6%)	38 (21.5%)	
Never quit	30 (10.4%)	11 (6.2%)	
Never drink	182 (63.0%)	128 (72.3%)	
Medical history			
Hypertension	162 (56.1%)	142 (80.2%)	<0.01
Diabetes	81 (28.0%)	35 (19.8%)	0.05
Prior MI	7 (2.4%)	9 (5.1%)	0.19
Prior stroke	16 (5.5%)	14 (7.9%)	0.33
Dyslipidemia	81 (28.0%)	73 (41.2%)	<0.01
Cancer history	5 (1.7%)	4 (2.3%)	0.74
AF	3 (1.0%)	4 (2.3%)	0.43

*MI, myocardial infarction; AF, atrial fibrillation*.

**Figure 4:**
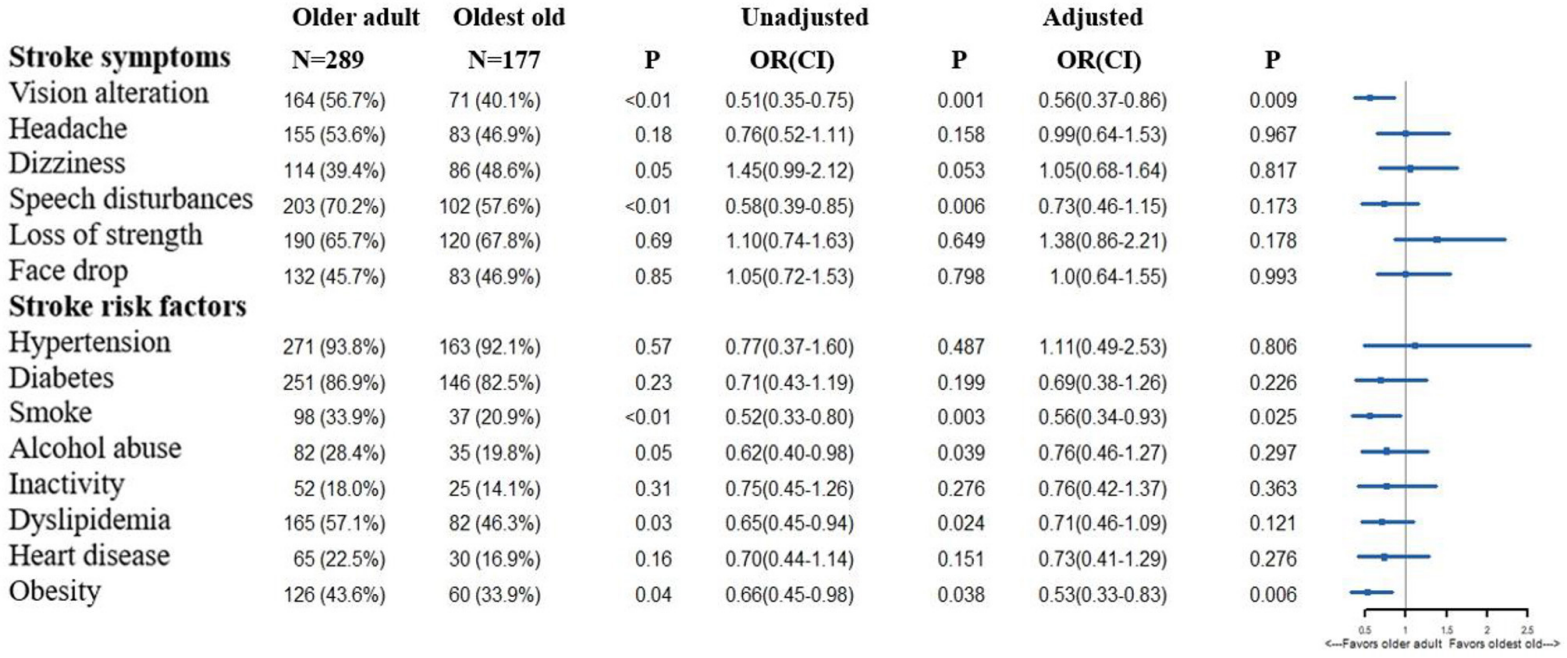
Differences in the knowledge of stroke symptoms and risk factors between older adults and the oldest old (age cut-off 75 years). OR, Odds Ratio; CI, Confidence Interval. In the multivariable logistic regression analysis, we adjusted gender, educational background, smoking history, drinking habit, hypertension, diabetes, myocardial infarction, prior stroke, dyslipidemia, cancer history, atrial fibrillation. The OR means the comparison between the older adults and oldest old (oldest old vs. the older adults).

**Figure 5:**
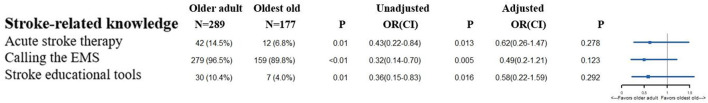
Differences in the awareness of stroke-related knowledge between older adults and the oldest old (age cut-off 75 years). OR, Odds Ratio; CI, Confidence Interval; EMS, emergency medical service. Acute stroke therapy included thrombolytic therapy and endovascular therapy. Stroke educational tools included FAST and Stroke 1-2-0. In the multivariable logistic regression analysis, we adjusted gender, educational background, smoking history, drinking habit, hypertension, diabetes, myocardial infarction, prior stroke, dyslipidemia, cancer history, atrial fibrillation. The OR means the comparison between the older adults and oldest old (oldest old vs. the older adults).

**Figure 6:**
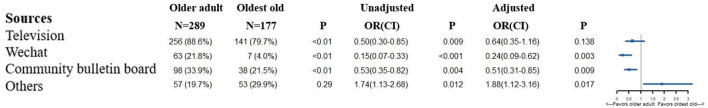
Differences in the sources of acquiring stroke-related knowledge between older adults and the oldest old (age cut-off 75 years). OR, Odds Ratio; CI, Confidence Interval. In the multivariable logistic regression analysis, we adjusted gender, educational background, smoking history, drinking habit, hypertension, diabetes, myocardial infarction, prior stroke, dyslipidemia, cancer history, atrial fibrillation. The OR means the comparison between the older adults and oldest old (oldest old vs. the older adults).

Although the between-group differences in the awareness of face drop, alcohol abuse, and dyslipidemia were non-significant in the multivariable logistical regression analysis, older adults showed a tendency of better awareness of stroke symptoms, risk factors, acute stroke therapy, and calling the EMS (Figures 4–6). Notably, regarding the sources of acquiring stroke-related knowledge, television lost significance in the multivariable logistical analysis, while the others became statistically significant. After checking the original data, the other sources included chatting with neighbors, reading newspapers, and talking with family physicians.

## Discussion

Population aging is correlated with an expected increase in the burden of cardiovascular disease (13). Previous studies on stroke knowledge among community adults reported the association of age with poor stroke awareness (5, 6). However, only a few of these studies investigated the awareness among older adults and compared stroke knowledge between older adults and the oldest old. We observed between-group differences in stroke knowledge. The older adults showed better knowledge of stroke symptoms and risk factors than the oldest old; moreover, there were considerable variances in the sources for acquiring stroke knowledge.

With respect to the awareness of stroke symptoms, the oldest old showed a tendency of lower awareness of vision alteration and face drop as an indication of stroke onset than older adults; however, the awareness of face drop lost significance when the cutoff age was 75 years. Older adults with advanced age often have vision problems that they are yet to be accustomed to, which could reduce the awareness of vision alteration as a stroke symptom. Moreover, the disparity in the results under the two different cutoff ages was also observed for the awareness of acute stroke therapy and calling the EMS. This difference could be attributed to several factors. First, among older adults aged >75 years, even a 5-year gap can result in significant differences in stroke knowledge, which could result from the lower educational level in the oldest-old group, even after adjusting for potential baseline confounders. Second, the number of older adults aged >80 years was small, which may be less representative. Regarding the awareness of stroke risk factors, a similar disparity between the cutoff values was observed. Awareness of smoking and obesity as stroke risk factors was consistently higher in older adults than in the oldest old. The underlying reason for this should be investigated in future studies. Our findings suggest that stroke educational campaigns should pay more attention to older adults with advanced age to eliminate differences in stroke knowledge, especially regarding the stroke symptom of vision alteration and the stroke risk factors of smoking and obesity. Additionally, differences in the sources of acquiring stroke-related knowledge indicated that the means of stroke knowledge promotion crucially affects the effectiveness of the campaign for community older adults.

From the literature review, our findings are consistent with those of a community-based questionnaire study (4), which included 2,519 participants from four Chinese cities (Beijing, Shanghai, Changsha, and Chengdu). In particular, arm/leg weakness was the most frequently known symptom, and hypertension was the most well-known risk factor of stroke. The awareness rate regarding other stroke symptoms except arm/leg weakness was 58.2–71.2%, which was higher than that of our findings, which could be attributable to younger participants (mean age: 55.72 years) being included in that study with ≥50% having completed primary school. Another study conducted in Chongqing, China, reported similar phenomenon of low awareness of stroke symptoms and risk factors among community residents (14), in which only 23.3% of the participants knew about thrombolytic therapy (15).

Studies from outside China have also revealed a low awareness of stroke symptoms and risk factors. Krzystanek et al. (16) showed that nearly 50% of respondents could not recollect any stroke symptom, whereas only 38.6% participants could list two or more risk factors, probably because their awareness was investigated by asking open-ended questions. On the contrary, Patel et al. (17) found high awareness of stroke symptoms by analyzing a large national health survey; the awareness rate was ≥70% for all five stroke symptoms. They also asked closed-ended questions like we did, so the differences may be attributable to the sociodemographic disparities. Surprisingly, Diez-Ascaso et al. (18) found that even for patients with prior stroke, only 15.6% acknowledged all their vascular risk factors, while 52.1% acknowledged some of them, and 32.3% failed to recognize any. Therefore, further efforts would be necessary to improve their knowledge on vascular risk factors.

Only a few studies targeted the older population, who are more vulnerable to stroke, especially with respect to aging worldwide. In India, an observational study conducted by Bhat et al. (19) reported low awareness of stroke symptoms and risk factors among elderly patients with hypertension. Specifically, ~40% of participants had never heard about the term “stroke” before. Another study in Argentina (20) reported that only 65% of 367 elderly adults would call the EMS when they experienced typical stroke symptoms. Khalil et al. (6) also found that in case of stroke suspicion, only 57.69% of adults aged 50 and above would call an ambulance in Lebanon. Although our findings indicated that ~90% of the older adults responded by calling the EMS after identifying a stroke, this finding should be interpreted with caution. This is because, in most cases, it is quite difficult for community residents to correctly identify stroke onset.

Oldest old adults considered television as an important source for acquiring general health-related knowledge, but not related to the awareness on stroke, and it lost significance when the cutoff age was 75 years. This indicates that older adults may not be interested in watching advertisements that discuss stroke-related knowledge. Therefore, Wechat or a community bulletin board may be more effective to educate them regarding stroke than television. Using those mediums could also reduce the expenses of a mass-media stroke campaign, which levies high costs. As the general clinical practice has developed in recent years, provisions of a family physician team have been allocated to each community in China. In the future, stroke physicians should collaborate with family physicians to promote stroke knowledge by these promising, yet cost-effective means.

## Limitation

This study has several limitations. First, our findings were yielded through a voluntary questionnaire. Although we attempted to ensure that the response rate was >90%, differences may still exist between the included and excluded participants. Second, we only included two suburban communities so the sample size was quite small in consideration of the 264 million people aged >60 years in our country. Moreover, the proportion of the oldest old was low, which may not be representative of all people with advanced age in China. There is a need for future larger cross-sectional surveys that include more communities across China. Third, the awareness rate of stroke knowledge among the older adults may have been overestimated due to the closed-ended design of the questionnaire. A previous study reported that when participants receive an open-ended questionnaire, >40% could name at least one of the five stroke symptoms included in SUDDEN; none of the participants could name all five symptoms. However, when warning signs were read from a list, almost all participants identified at least one symptom; furthermore, ~50% of the participants identified all five symptoms (21). This suggests that our findings could considerably vary depending on how the questions were answered. Fourth, We have not thought about the influences of cognitive impairment on stroke knowledge among community older residents. After talking with community physicians, of the 20 participants who cannot finish the survey and were accompanied by their children, 12 of them were diagnosed with cognitive impairment, two of them were diagnosed with dementia, and six of them with difficulty in hearing. Of those participants included in this study, we have not performed cognitive test on them, so the influences cannot be evaluated. However, based on the training on community physicians before the program, these kinds of influences should have been minimized since stroke physicians had reminded them to exclude participants with obvious cognitive decline in the training session. Older adults with prior stroke are more likely to have cognitive impairment, and the proportion of participants with prior stroke only accounts for 6.44% in our study, so cognitive decline may only have limited impact on the awareness of stroke-related knowledge in this study.

## Conclusion

With the aging of the population across China, the life expectancy is expected to be longer in future decades. There is a need for sufficient stroke knowledge to avoid the adverse outcomes of stroke, especially in older adults. On one hand, we should encourage the oldest old to participate in stroke educational campaigns. On the other hand, there is a need for special efforts to address the disparities in stroke knowledge between older adults and the oldest old.

## Data Availability Statement

The raw data supporting the conclusions of this article will be made available by the authors, without undue reservation.

## Ethics Statement

The studies involving human participants were reviewed and approved by Institutional review board affiliated to Fudan University. The patients/participants provided their written informed consent to participate in this study.

## Author Contributions

XL conceptualized and designed the study, handled the supervision and coordination, and revised the manuscript. HG also designed the study, handled the supervision and coordination, and revised the manuscript. SY was in charge of the acquisition of data and the raising of funds. ZL also did the acquisition and interpretation of the data. JZ also contributed in the conceptualization and design of the study, supervision and coordination, fund raising, and revising the manuscript.

## Conflict of Interest

The authors declare that the research was conducted in the absence of any commercial or financial relationships that could be construed as a potential conflict of interest.

## Publisher's Note

All claims expressed in this article are solely those of the authors and do not necessarily represent those of their affiliated organizations, or those of the publisher, the editors and the reviewers. Any product that may be evaluated in this article, or claim that may be made by its manufacturer, is not guaranteed or endorsed by the publisher.
